# Frequency of Rotavirus and Adenovirus Gastroenteritis Among Children in Shiraz, Iran

**DOI:** 10.5812/ircmj.4415

**Published:** 2013-08-05

**Authors:** Mohammad Motamedifar, Elham Amini, Pedram Talezadeh Shirazi

**Affiliations:** 1 Shiraz HIV/Aids Research Center (SHARC), Medical Faculty, Shiraz University of Medical Sciences, Shiraz, IR Iran; 2 Department of Bacteriology and Virology, Medical Faculty, Shiraz University of Medical Sciences, Shiraz, IR Iran

**Keywords:** Rotavirus, Adenovirus, Gastroenteritis, Child

## Abstract

**Background:**

Viral pathogens are the main cause of acute gastroenteritis in developed and developing countries. Rotavirus and adenovirus are the two important agents associated with hospitalization for diarrhea especially in children. Limitation and control of diarrhea as a costly disease must be considered in national health programs.

**Objectives:**

Epidemiological studies on viral diarrhea and collecting data for rotavirus and adenovirus prevalence, as two important viral agents of gastroenteritis, are valuable for planning of a prospective program.

**Materials and Methods:**

827 stool samples of pediatrics patients with gastroenteritis who were admitted to Dastgheib Hospital, Shiraz, Iran, from September 2008 to February 2010 were tested for presence of rotavirus and adenovirus using the EIA method. A demographic and clinical study was performed to determine the relationship between viral infection and clinical outcomes of patients.

**Results:**

Rotavirus was identified in 347 patients out of 827 (42%), adenovirus was detected in 76 (9%) of samples and 34 (4%) of patients had rotavirus-adenovirus co-infection. Diarrhea was the most common symptom in viral infected patients.

**Conclusions:**

Given the non-specific symptoms of these viruses and the high prevalence of viral diarrhea in our region, more laboratories should be equipped for virus detection and vaccination might be considered as a prevention strategy.

## 1. Background

Acute gastroenteritis is the most common cause of morbidity and mortality throughout the world. Although all bacteria, viral agents and parasites could be responsible for gastroenteritis, many studies have illustrated that viral agents are the most important factors (1-3). In spite of the improving sanitation in many countries, some viral infections have not been eradicated. Improving socio-economic conditions and collecting data or reports on viral infections in different countries are necessary for decision making health policies (4). Among viral agents of gastroenteritis, rotavirus is known to be one of the frequent etiologic agent of viral diarrhea leading to a burden of many direct and indirect costs, such as physician visits and hospitalization and missing work, all over the world(5, 6). Rotavirus is transmitted via the feco-oral route and, following establishment in the small intestine, results in nutrient malabsorption (7). The clinical spectrum of acute rotavirus gastroenteritis ranges from a self-limited watery diarrhea illness accompanied with nausea, anorexia and mild vomiting or fever, to sever dehydration resulting in hospitalization or even death (8). Diarrhea, vomiting and fever are the most common presentations. Although rotavirus primarily infects children, it could cause a mild disease in adults. Since 95% of children experience rotavirus infection at the age < 5years, the lower rate of rotavirus gastroenteritis in adult seems to be due to the presence of antibody against the virus. Also, presence of maternal antibody in neonates at the age < 3months leads to a reduction in severity of disease during the first month of life (9, 10). The prevalence of rotavirus gastroenteritis is also variable in different seasons and months and is mostly observed in winter. In some reports, adenovirus is considered as another etiological agent of viral gastroenteritis after norovirus, rotavirus and astrovirus (11, 12). Adenoviruses are non-enveloped, ds DNA viruses with 52 recognized serotypes. Its infection is mostly seen in pediatrics up to 2 years old. The serotypes 40 and 41 enteric adenoviruses are associated with gastroenteritis in pediatrics. Investigations on gastroenteritis prevalence illustrated that the incidence of enteric adenovirus infection is nearly 3 times greater in developing countries than developed ones (3, 13).

## 2. Objective

Due to the lack of recent reports on surveillance of rotavirus and adenovirus infection in Shiraz, a major city in southwest of Iran, this study was undertaken to determine the frequency and clinical significance of the aforementioned viruses in children with gastroenteritis in Shiraz.

## 3. Materials and Methods

### 3.1. Study Design

A total number of 827 stool samples were collected from children exhibiting symptoms of diarrhea and/or vomiting admitted to Dastgheib Hospital, from September 2008 to February 2010. This hospital was established since 1950 in Shiraz, Iran and is the major pediatric referral center with about 80000 annual admissions. The fecal specimens were divided into two aliquots immediately, one of which was stored at -70°C for later virological tests. The patients' demographics, chief complaints at referral, associated symptoms and clinical findings such as abdominal pain, lethargy, fever, vomiting and diarrhea noted by the physicians were screened from the patient files.

### 3.2. Virus Detection

For the detection of rotavirus antigens in fecal samples, enzyme immunoassays were performed using ROTASCREEN II® EIA kits (Microgen Bioproducts Ltd, UK) with 98.6% sensitivity and 100% specificity. ADENOSCREEN®EIA kits (Microgen Bioproducts Ltd, UK), with 98% sensitivity and specificity, were used for adenovirus detection. Enzyme immunoassays were performed according to the manufacturer's instructions.

### 3.3. Statistical Analysis

Statistical analyses were performed using SPSS v.16 software. Descriptive statistics are shown as percentages and ratios. Chi square (χ2) test was used to compare groups. Probability P < 0.05 was regarded as statistically significant. Multivariate analysis was performed using a logistic regression method. In this test positive virus was used as the dependent variable and other clinical findings were used as independent variables. The test was used for each virus separately.

## 4. Results

Of the 827 children admitted with gastroenteritis, 492 (59.5%) were male and 40.5% were female. The patient age ranged from 0 to 144 months, with a mean of 18 months. The descriptive statistics illustrate that rotavirus was identified in 347 patients out of 827 (42%). Adenovirus was detected in 76 (9%) of samples and 34 (4%) of patients had rotavirus-adenovirus co-infection. There was no significant difference between rotavirus or adenovirus positivity between genders. However, the rate of positive rotavirus and adenovirus was higher in male cases (62% for positive rotavirus and 64% for positive adenovirus cases). But co-infection rate was three times higher in male patients than the females. In other words, 27 of 34 positive co-infected cases were male (P = 0.016).

**Figure 1.**
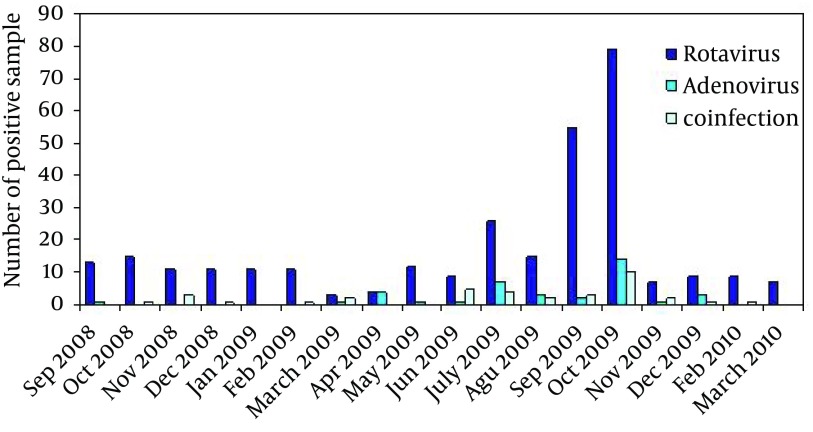
Distribution of Virus-Positive Patients in Different Age Groups

Figure 1 shows the distribution of virus positive patients by the age range of patients. The patients ranged 6-17 months old had the highest infection rate for the viruses (P < 0.05). However, the rate of co-infections did not differ significantly in different age groups (P = 0.436). 

**Figure 2.**
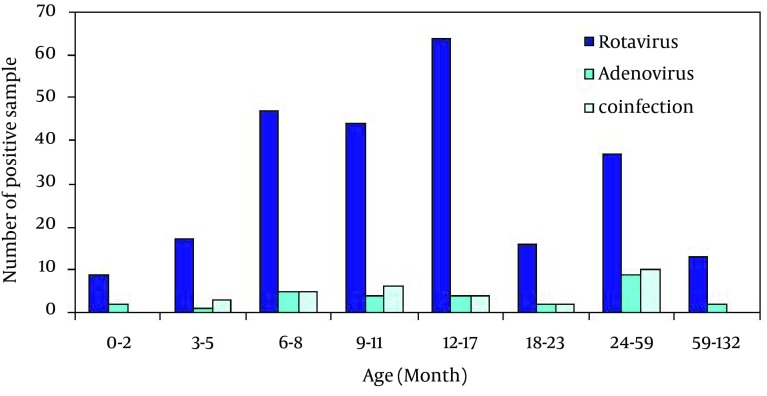
Distribution of Rotavirus Infection, Adenovirus Infection and Co-Infection by the Month of Admission

The distribution of rotavirus infection, adenovius infection and co-infection by the month of admission are depicted in Figure 2. The number of virus positive cases was significantly different in respect to the date of admission to the hospital. Totally 175 (51.3%) of rotavirus positive samples were detected in just two months of September and October 2008 and 2009. Also higher number of adenovirus positive samples (48.7%) was isolated in July and October of 2009. However, the rate of rotavirus-adenovirus co-infection was nearly 29% (10 case of 34) in October of 2009, and there was no meaningful statistically difference in respect to the month of referral (P > 0.05). 

With regard to clinical parameters, 82% of rotavirus positive patients and 51% of adenovirus positive patients had diarrhea. The rate of diarrhea was 91% in co-infected patients. Vomiting was determined in 77% of rotavirus infected patients, 66% of adenovirus infected patients and 64% of co-infected cases. However the presence of vomiting was not significantly different between positive and negative cases for rotavirus (P = 0.420), adenovirus (P = 0.051) and co-infection (P = 0.097). Fever was observed in 73% of rotavirus positive cases, 61% of adenovirus infected patients and 62% of co-infected patients.

Logistic regression analysis, as the most convenient method for testing the usefulness of predictor variables, illustrated that diarrhea was the best indicator regarding absence or presence of rotavirus in patients. In adenovirus patients there was a significant relationship between adenovirus infection and three parameters of diarrhea, fever and coincidence of fever and diarrhea. However, diarrhea showed the most powerful relationship with adenoviral gastroenteritis and fever was in the second position. No significant association was found between vomiting, abdominal pain, lethargy, PMN in stool, blood in stool and nausea with rotavirus and/or adenovirus infection. The severity of disease between these two types of viruses was not different. In rotavirus-adenovirus co-infections, like rotavirus infections, diarrhea was the most important predictor variable.

## 5. Discussions

Roatvirus and adenovirus are important causes of viral diarrhea in many countries and have led to noticeable deaths in children in the past decade (1). However, in the past other viruses such as noroviruses and astroviruses were accounted for the many of documented gastroenteritidis viral infections in some developed countries (11). In any case, diarrhea as a costly disease has a great impact on the government budget and studying the causative agents including viruses are still necessary for the health policy decision makers. On the other hand, neglecting on the implementation of the virus detection tests in clinical laboratories leads to longer detection procedures and in many cases, redound to antibiotic therapy (4). From this point of view, viral screening for rotavirus and adenovirus especially for children under 5 years seems to be necessary.

In this survey, 347 (42%) positive rotavirus cases were detected out of 827 children. The significant differences between rotavirus positive and different age groups are in good agreement with other reports from UK, Indonesia and Nigeria (3, 10, 14). The rate of rotavirus positivity in children aged less than 14 months was 53%. This finding is higher than those reported for Turkey with 21% 13and 14% (15), Southern Korea with 25% (16),and Nigeria with 15.6 % positivity (9) but is lower than the reported data for northern Iran (62%) (17).

In the present study, rotavirus was prevalent throughout the year with higher frequency in September and October similar to what was reported from Turkey (18) whereas in northern Iran, rotavirus infection incidence was higher in winter (68%) and autumn (62%) (17). Close geographical conditions of Iran and Turkey may be a factor for this similarity in rotavirus outbreak time, however, there are previous reports showing rotavirus outbreaks in different geographical locations US and Europe. In the United States, there are reports of a seasonal pattern for rotavirus prevalence which begins from the southwest in November and reaches to the northeast in April or May (1). In Europe, rotavirus is prevalent during January–March. In tropical countries such as Malaysia, a seasonal pattern for rotavirus was not reported (13).

Overall, adenovirus was detected in 9% of fecal samples whereas in the north of Iran it was 2.3% (17). Similarly, in different countries, a lower rate of adenovirus infection (compared to rotavirus) was reported. Adenovirus infection rate was reported as 14% by Topkaya et al. (15) and 8.9% by Akan (13) in Turkey and a range of 2-31% in developing country by Wilhelmi et al. (19). In our study, it was detected in a range of 1-11% in different age groups which was more frequent in children under 14 months old (11%). This finding is similar to the rates reported by Kheyami (20) and Barnes (21) from Australia. But this data is in contrast with 41.2% adenovirus positivity reported by Carraturo (22) which was also more frequent among children with 24-36 months in Italy. Also, in the study of Lin et al. (23) in Taipei 76.6% of children younger than 2 years were adenovirus positive.

In this study, we noted a peak in frequency of adenovirus in July and October. This peak coincided with the rotavirus peak in October as well. In previous reports adenovirus was detected throughout the year and there was no seasonal pattern or any peak in frequency of adenovirus through the year (13, 24). Rotavirus-adenovirus co-infection rate in this survey was 4%. More frequent co-infection was seen in October by the rate of 29% of all co-infections. Co-existence of more than one agent is not rare in gastroenteritis cases. This rate is similar with findings of Akan and Parashar (13, 25). Different rates of such co-infection in other countries were reported in Italy (1.3%) and in Turkey (8%) (15, 23).

In this study, the rate of viral co-infection was three times higher in male cases compared to females, however, no statistical difference was observed between the two genders. In some studies it was reported that the higher rate of viral gastroenteritis was seen in males and similarly this difference was usually insignificant (10, 15, 26, 27). Due to the logistic regression results, diarrhea was the main clinical feature of viral gastroenteritis among rotavirus and/or adenovirus positive patients in the present study. Also, significant relationships between adenovirus infection and three parameters of diarrhea, fever and coincidence of fever and diarrhea were found in this study. So, fever could be beneficial in distinguishing between rotavirus and adenovirus infections. We did not find any relationship between fever and rotavirus infection. Similar results were reported by Akan et al. (13). However, in some previous studies, fever was associated with rotavirus infection (28, 29).

Overall, according to the relatively high prevalence of viral gastroenteritis observed in this study and nonspecific clinical presentation, the importance of laboratory detection is obvious. Some easy tests such as ELISA, Latex agglutination and PCR have provided rapid diagnosis, but most of laboratories avoid conducting viral detection tests because of costly tests or absence of general guideline for viral diagnosis procedure (12, 30, 31). Updating the data of main viral agents of gastroenteritis, including rotavirus and adenovirus prevalence in developing countries are essential for the arrangement of a preventive strategies including national immunization programs. This study was carried out to partly address the limited published data in this field in different geographical regions of Iran. The results of this study emphasized the necessity of a prospective strategy for such an immunization planning program.
